# The After-Treatment of Tracheotomy Cases for Membranous Croup, with an Observation of Three Successful, out of Four Operations*Read before the Georgia State Medical Association, Augusta, Ga., April 16, 1896.

**Published:** 1896-05

**Authors:** R. M. Harbin

**Affiliations:** Rome, Ga.


### THE AFTER-TREATMENT OF TRACHEOTOMY CASES FOR MEMBRANOUS CROUP, WITH AN OBSERVATION OF THREE SUCCESSFUL, OUT OF FOUR OPERATIONS.*

By R. M. HARBIN, M.D.,

ROME, GA.

It might be proper to preface my remarks on the after-treatment of tracheotomy cases for croup with a brief discussion of the etiology, symptoms, diagnosis, and the medicinal treatment of membranous and diphtheritic croup.

The most recent text-books have ceased to give space to membranous croup as a disease distinct from diphtheria. While it is safe to regard membranous and diphtheritic croup as the same disease in densely populated districts, yet I believe there are many

*Read before the Georgia State Medical Association, Augusta, Ga., April 16, 1896.

cases of membranous croup in isolated localities where diphtheria or other infectious diseases are practically unknown.

My first case of tracheotomy for croup was in a country district, in a family where there were five other children, who were all healthy at the time, and no sickness of any kind was observed either six months before or after the time of the operation, and no attempts at isolation were made.

The incubative period of diphtheria varies from one to twenty days. My classmate, Dr. J. S. Stribling, of Seneca, S. C., writes me that he was called to see a child, suffering with diphtheria, at 5 A.M., and in attempting to detach a portion of membrane from the tonsil, some sputa was projected into his face and mouth. At 3 P.M. he was taken with a severe chill, and all the constitutional symptoms of diphtheria; thus making the incubative period ten hours. He had the diphtheritic sequela and recovered sufficiently to return to his practice only after twelve months.

The grandmother of the child upon whom tracheotomy (my fourth case) was performed for diphtheritic croup came from the country to visit the grandchild, with her own three-year-old child, on the fourth day after the operation. Six days later the child was taken with coryza, sore throat, swollen glands in the neck, erythematous rash over the back, etc., and returned to its home the next day. The second day after its return the child died with croup; thus making the incubative period about six days.

There are two forms of diphtheria. First, the pseudo-membranous inflammations, due to the Klebs-Loeffler bacilli; and, second, the varieties of diphtheria due to a streptococcus.

The Klebs-Loeffler bacilli are found only at the site of infection, and the systemic effects are due to the absorption of toxalbumen or poisonous products of the bacilli. While bacteriological examinations will do much towards clearing up the diagnosis, yet there are many cases where the bacilli cannot be found, and we are forced to depend on the clinical symptoms to make a diagnosis.

From a clinical point of view, membranous croup occurs in children from two to five years old, being rare in young infants, and usually occurs in isolated cases, and is not contagious. It is gradual in development and slightly paroxysmal in character and occurs without either pre-existing pharyngeal symptoms, constitutional depression, or glandular involvement, and has no sequela.

Diphtheria, on the other hand, begins in the pharynx, and, in the majority of cases, does not extend to the larynx, and has the constitutional symptoms of an infectious disease. It occurs along with other cases, and is contagious. Albuminuria is diagnostic of diphtheria. The Klebs-Loeffler bacillus, when found, makes the diagnosis positive.

The medical treatment includes a great variety of drugs which should be tried, if possible, before resorting to surgical treatment. The remedies that have been recommended for croup are too numerous to mention, and I will only speak of a few that I have found to give temporary relief. Calomel by sublimation may be used, and fifteen grains every two hours is the quantity usually employed. Vaporized inhalations of lime water and turpentine along with cold or hot applications to the neck may also be tried. Turpeth mineral occasionally gives relief.

It is very exceptional for a case of membranous laryngitis to recover from medicinal treatment alone, and we are driven to the consideration of the surgical treatment.

Opinions differ as to the relative value of intubation and tracheotomy, but the percentage of recoveries from statistics is about the same in both instances, being slightly in favor of intubation. From a collection of twenty-one hundred and eighty-three cases of tracheotomy by Lovett and Munroe, twenty-eight per cent. of recoveries is given, and from a collection of forty-two hundred and seventeen cases of intubation from all sources, about thirty per cent. of recoveries is reported. The statistics are misleading in that many mild cases are intubated, whereas tracheotomy is done only as a last resort. The only advantage that I can see that intubation has over tracheotomy is, that the parents assent to its use early in the disease and the case can finally be submitted to tracheotomy. The great desideratum in both operations is to admit air into the lungs and to free the trachea and the bronchi of the retained sputa. Intubation is by far inferior to tracheotomy in fulfilling the latter indication, and the prospects for recovery can be estimated by the amount of expectoration.

After having given medicinal treatment a fair trial and the dyspnea progressively gets worse with beginning cyanosis, resort immediately to tracheotomy, for the exceptional cases that recover without operative interference at this stage of the disease are not to be considered. In a majority of cases a sufficient time is had to give the various remedies a fair trial.

Where laryngeal obstruction is rapidly developed, the formation of membrane in the trachea, with an extension upwards, is probable, and this class of cases is not so amenable to tracheotomy.

The instruments needed are not numerous, and only a silver tube in addition to the general operating instruments is required.

An anesthetic should be used very cautiously, and chloroform is preferred, as quietude of the patient is absolutely essential to a rapid operation. A child with croup makes a very rebellious patient when in the last stages of the disease.

The superior is preferable to the inferior operation, as the trachea is more easily exposed in the former. The ring of the cricoid cartilage is the guide, and from this point an incision should be made downwards, two and a half inches long, the larynx being steadied with a tenaculum.

A long incision makes the operation more easy to expose the trachea, and this is one of the most important details of the procedure. Dissection should be made with the point, or the handle of the scalpel pushing aside any vessels that may appear and the lobes of the thyroid gland.

The trachea may be opened from below upwards with a small scalpel resting on the palmar surface of the right index finger, which serves as a guide. The slit may be hooked up with an aneurism needle and the tube is introduced.

If the skin incision has been very extensive it may be sutured with silk and the tube must be secured by tapes around the neck. A feather should be dipped in lime-water and introduced into the tube to stimulate cough and expectoration.

The after-treatment is the most important part of the whole procedure, and I attribute the favorable results of my cases almost entirely to this part of the treatment.

The essential part of the after-treatment was the persistent use of lime-water and maintaining the room temperature at sixty degrees to seventy degrees F. The feathers used should be of proper size and stiffness, and should be soaked in bichloride solution before using them. The tube should be moistened every few seconds, night and day, by dipping the feather in lime-water, thus allowing it to trickle down the tube. I believe lime-water to be an excellent solvent, and, if used too freely, it is only coughed out, making the expulsion of shreds of membrane and tenacious mucus more easy. The nurse can be directed to use it to the extent of producing a bubbling sound in the tube nearly all the time, and occasionally introduce the feather down into the windpipe to stimulate cough. When the excess of lime-water is coughed up, some of it is projected along the sides of the tube to the vocal cords, thus effecting disintegration of the membrane. This part of the treatment can be done continuously without disturbing the patient, and almost any nurse can very soon learn the indications to be met, and should be ever ready to remove the sputa when once coughed up, to prevent its being drawn back through the tube. Vaporized solutions for inhalation fulfill a good indication, but the apparatus necessary are usually inconvenient to be had outside the hospitals.

The inner tube should be removed every few hours and washed in a soda solution. Iodoform ointment should be applied every day to the edges of the wound, as the dried sputa forms such hard crusts that it annoys the patient. The chest should be rubbed with camphorated oil and enveloped in flannel every day. Whisky should be given as eggnog, milk punch, or toddy, but if patient refuses, it should be given per rectum when necessary.

A liquid diet, including milk, broth, coffee, etc., should be given freely.

As the case advances expectoration becomes more liquid and easy, so that about the tenth day the sputum has lost its purulent qualities and the tube may now be permanently removed.

After the fourth day the whole tube should be removed and washed and a moist pad applied to the wound so as to encourage breathing through the larynx. Tracheotomy keeps the patient alive until the membrane is disintegrated by the lime water and furnishes a means of expectorating the débris.

The causes of death after tracheotomy are septicemia, diphtheritic poison, heart clot, and diphtheritic paralysis.

The patient who survives the end of the third day will probably get well.

After the removal of the tube the edges of the wound should be kept in apposition with adhesive plaster applied in the following manner: Make two rolls of cloth about one-sixth of an inch in diameter and one inch long and place them on either side of the wound. Over these the plaster is applied every day for the first two or three days. The advantage of this is, the bottom edges of the wound are first approximated and the opening heals up usually in five to seven days, and voice returns to normal in about two weeks.

I have four cases to report, three of which recovered, and in all the operation was done as a last resort; so the favorable results cannot be attributed to early operations.

I regret that I have not the bacteriological examinations of the cases to report, and consequently only clinical diagnoses were made. In none of the cases were there the characteristic diphtheritic membranous deposits observed in the pharynx, or on the tonsils. Two of the cases were diagnosed diphtheritic, and the two remaining cases were non-diphtheritic. In both of the latter the result was favorable.

Case 1. E. H., a healthy, four-year-old girl, living in the country, suffered with croup three days. The dyspnea had gradually developed, and at the time of the operation the patient was cyanotic and in a semi-comatose condition, with a pulse of one hundred and sixty. The diagnosis of membranous croup was made. After having tried all the remedies that could be thought of without any success, tracheotomy was done, September 23, 1891, at 6 P. M. Expectoration was abundant, and the child made a good recovery, and has been well ever since.

Case 2. C. D., a boy three years and two months old, was taken with hoarseness four days previous to the time I saw him and had developed aphonia with excessive dyspnea. Membranous croup was diagnosed and tracheotomy was done as a last resort, January 9, 1893, at 4 P. M. For the first twenty-four hours expectoration was scanty and temperature rose to one hundred and four degrees F., but by the persistent use of lime-water expectoration became more loose, and the fever was reduced. The patient made a good recovery and has been well ever since.

CASE 3. E. H., a delicate and nervous boy, seven years old, was taken with hoarseness March 8, 1893, at 8 A. M. Dyspnea suddenly developed at 3 P. M., with marked cyanosis, and pulse was reduced to forty, and was barely perceptible. Diphtheritic croup was diagnosed, as a younger brother died a few days previous with diphtheria, complicated by pneumonia. The patient reacted very slightly and tracheotomy was very hurriedly done with very unfavorable surroundings. Expectoration was absent and dyspnea was only partially relieved on account of the formation of the membrane in the trachea. The patient died thirty hours later in a convulsion.

CASE 4. R. G., a two-and-a-half-year-old boy was taken with hoarseness and croup, December 2, 1895, at 9 P. M. I was called to see the child with Dr. D. H. Ramsaur, December 6, at 1 P. M. The diagnosis of diphtheritic croup was made and dyspnea was now extreme. All the various remedies were tried with no success and at 4 P. M. tracheotomy was resorted to in the presence of Drs. Ramsaur and Wynn. The patient reacted well and expectorated freely, by using the lime-water as previously indicated. I might mention incidentally that the patient vomited a worm six inches long on the third day after the operation. The tube was removed on the eleventh day and recovery was complete.

Conclusions:

First. Croup, whether diphtheritic or membranous, is almost invariably fatal without surgical treatment, and the few cases that recover by medicinal treatment alone are not to be considered.

Second. So far as the practical indications for tracheotomy are concerned, it makes no difference whether the croup be diphtheritic or membranous.

Third. Tracheotomy has the advantage over intubation, in that it gives a better means of expectorating the membranes and furnishes free drainage from the sight of septic infection.

Fourth. Tracheotomy is a justifiable surgical precedure, and should be performed in all cases where our therapeutic resources have been exhausted and where the patient is in imminent danger of suffocation.

It should be done in hopeless cases, since it either offers a chance for the patient or promotes euthanasia.

Fifth. Tracheotomy keeps the patient alive until the pseudo-membrane disintegrates and resolves into a muco-purulent liquid and is expectorated through the tube.

Sixth. The after-treatment is the most important part of the procedure, and I attribute the successful results reported to the persistent use of lime-water as previously outlined.

